# Temporal Trends in the Prevalence of Diabetes Decompensation (Diabetic Ketoacidosis and Hyperosmolar Hyperglycemic State) Among Adult Patients Hospitalized with Diabetes Mellitus: A Nationwide Analysis Stratified by Age, Gender, and Race

**DOI:** 10.7759/cureus.4353

**Published:** 2019-04-01

**Authors:** Rupak Desai, Sandeep Singh, Muhammad Haider Syed, Hitanshu Dave, Muhammad Hasnain, Daniyal Zahid, Mohammad Haider, Syed Muhammad Ali Jilani, Muhammad Ali Mirza, NFN Kiran, Ali Aziz

**Affiliations:** 1 Cardiology, Atlanta Veterans Affairs Medical Center, Decatur, USA; 2 Clinical Epidemiology, Biostatistics and Bioinformatics, Academic Medical Center University of Amsterdam, Amsterdam, NLD; 3 General Surgery, Sindh Government Hospital, Karachi, PAK; 4 Internal Medicine, Hackensack Meridian Health Jersey Shore University Medical Center, Neptune City, USA; 5 Surgery, Jiujiang University Medical College, Jiujiang, CHN; 6 Internal Medicine, Robert Wood Johnson University Hospital, New Brunswick, USA; 7 Internal Medicine, Newyork-Presbyterian Brooklyn Methodist Hospital, Brooklyn, USA; 8 Endocrinology, LA Biomed, University of California Los Angeles Medical Center, Torrence, USA; 9 Internal Medicine, Frontier Medical & Dental College, Abbottabad, PAK; 10 Public Health, Emory Rollins School of Public Health, Decatur, USA; 11 Hospitalist, Covenant Medical Center, Waterloo, USA

**Keywords:** diabetes mellitus, decompensation, diabetic ketoacidosis, hyperosmolar hyperglycemic state, myocardial infarction, age gender racial disparities, trends, mortality, stroke

## Abstract

Background

Disproportionate change in the burden of diabetes mellitus across various subgroups has been reported in the United States. However, changing landscape of the prevalence and mortality of decompensated diabetes (diabetic ketoacidosis (DKA) and hyperosmolar hyperglycemic state (HHS)) remains indistinct across various age, gender, and racial groups of hospitalized diabetics.

Methods

The National Inpatient Sample (NIS) datasets (2007-2014) were sought to assess the prevalence and temporal trends in decompensated diabetes stratified by age, gender, and race and related in-hospital outcomes among the adult patients hospitalized with diabetes using International Classification of Diseases, Ninth Revision, Clinical Modification (ICD-9-CM) codes. Discharge weights were used to obtain national estimates.

Results

Of 56.7 million hospitalizations with diabetes, 0.5 (0.9%) million patients revealed decompensated diabetes from 2007 to 2014. The decompensated diabetics consisted of younger (~52 vs. 66 yrs), more often black (24.2% vs. 17.3%) and Hispanic (12.9% vs. 10.9%) patients as compared to those without decompensation (p<0.001). Younger diabetes patients demonstrated the highest prevalence of in-hospital decompensation (18-44 yrs; 3.7%) with a relative increase of 32.4% (from 3.4% to 4.5%) from 2007 to 2014 (p_trend_<0.001). Older diabetics (≥65 years) with decompensation suffered the highest in-hospital mortality (12.8%). The overall rate of decompensation was similar (0.9%) among male and female diabetes patients. However, over a period of 8 years, the rates of decompensation rose to 1.1% (p_trend_<0.001) in males and 1.2% (p_trend_<0.001) in females, respectively. All-cause in-hospital mortality among females with decompensated diabetes declined from 6.6% in 2007 to 5.9% in 2014 (p_trend_=0.019). However, there was no significant drop in in-hospital mortality among male diabetics with acute decompensation (6.7% in 2007 to 6.8% in 2014, p_trend_=0.811). We observed significantly increasing trends in decompensated diabetes among all race groups between 2007 and 2014 (p_trend_<0.001). The in-hospital mortality was highest among Asian or Pacific Islander (0.9%) diabetes patients with decompensation from 2007 to 2014. There was a declining trend in the inpatient mortality among Asian or Pacific Islander (p_trend_=0.029) and Hispanic (p_trend_<0.001) patients with decompensated diabetes, whereas other race groups did not observe any significant decline in mortality over the study period. Diabetes hospitalizations with decompensation demonstrated significantly higher in-hospital mortality (6.3% vs. 2.6%; p<0.001), average length of stay (7.7 vs. 5.4 days; p<0.001), hospital charges ($65,904 vs. $42,889, p<0.001), and more frequent transfers to short-term hospitals (3.9% vs. 2.9%; p<0.001) in comparison to those without decompensation. The rates of acute myocardial infarction (AMI) (10.4% vs. 4.8%; p<0.001), stroke (4.0% vs. 3.3%; p<0.001) and venous thromboembolism (3.5% vs. 2.6%; p<0.001) were substantially higher among diabetics with decompensation compared to those without.

Conclusions

There was an increasing trend in the prevalence of decompensated diabetes from 2007 to 2014, most remarkable among younger black male diabetics. The patients with decompensated diabetes suffered higher in-hospital mortality and rates of AMI, stroke and venous thromboembolism, there was no significant decline in the mortality between 2007 and 2014.

## Introduction

An alarmingly increasing prevalence of diabetes has been reported among adult population in the United States (US) between 1988 and 2012 [1]. Diabetes-related morbidity and mortality have caused an estimated loss of $245 billion to the US infrastructure in 2012 [2]. Decompensated diabetes is an umbrella term for diabetic ketoacidosis (DKA) and hyperosmolar hyperglycemic state (HHS), the two grave complications of diabetes.

DKA is a fatal manifestation reported in nearly 25-30% of patients with type 1 diabetes and in 4-29% of younger type 2 diabetics [3]. Limited data exist on the prevalence of HHS with a comparatively lower incidence rate than DKA and is estimated to be less than 1% of all diabetes-related hospitalizations [4]. However, the mortality rate of HHS is significantly (~10 times) higher compared with DKA [4]. DKA occurs more commonly in type 1 diabetics and younger type 2 diabetics, while HHS occurs predominantly in the elderly population [3-5]. However, an increasing prevalence of DKA has been reported among younger type 2 diabetics. An overall rising trend in the DKA-related hospitalizations has been described in the past decade in the US [3]. However, it remains poorly defined if these patterns fluctuate by various age, sex, and race groups of the hospitalized diabetic population.

Younger age and obesity are known risk factors for decompensation among diabetics. The increasing prevalence of obesity over the past few decades remained consistent even with a growing trend in diabetes [6-7]. However, the rate of increase in DKA has been perceived to be more rapid in comparison to the overall increase in diabetes [8]. The disproportionately changing landscape of demographics and risk factors of the US population could have an impact on the diverse burden of DKA/HHS across various subgroups. Since age, gender, and racial inconsistencies in the healthcare services accessibility have been widely accounted for in the US, it is imperative to comprehend the changing pattern of DKA/HHS and related outcomes across these subgroup populace. Therefore, the main objective of the present study is to analyze the trends in the incidence of decompensated diabetes among all diabetes-related admissions stratified by age, gender and race and the impact of acute decompensation on in-hospital outcomes using the largest nationwide cohort of adult diabetics in the US from 2007 to 2014.

## Materials and methods

Data sources

The administrative data from 2007 to 2014 were acquired through the Healthcare Research and Quality Healthcare Cost and Utilization Project-sponsored National Inpatient Sample (NIS) datasets [9]. The NIS remodeled its sampling strategy using self-weighing design in 2012 to improve national evaluations. Each year the NIS consists of a sample of 8 million unweighted or 35 million weighted hospital discharges. Prior to 2012, the NIS examined approximately all hospitalization records from 1000 hospitals (a 20% hospital sample). Beginning with the 2012 data year, the NIS approximates a 20% stratified sample of all discharges from U.S. community hospitals, excluding rehabilitation and long-term acute care hospitals. The NIS is sampled from the State Inpatient Databases (SID), which include all inpatient data that are currently contributed to HCUP and is representative of >95% of the US population. This study was exempted from the Institutional Review Board (IRB) review as it contained deidentified discharge data.

Study population

We identified diabetes decompensation (DKA/HHS) among diabetes-related hospitalizations using previously validated International Classification of Diseases, Ninth Revision, Clinical Modification (ICD-9-CM) codes: DKA (ICD-9-CM codes 250.10-250.13) or HHS (ICD-9-CM codes 250.20-250.23) also detailed elsewhere [10]. The baseline demographics, hospital-level characteristics, and pre-existing comorbid conditions were assessed between diabetes patients with vs. without decompensation. Temporal trends in diabetes decompensation and related in-hospital mortality were assessed for different age (18-44, 45-64, ≥65 yrs), gender (male and female) and race (white, black, Hispanic, Asian or Pacific Islander, Native American, others) groups.

Outcomes

The primary outcomes were trends in diabetes decompensation and subsequent in-hospital mortality among adult diabetes-related hospitalizations stratified by age, gender, and race. Co-primary outcomes were in-hospital complications which included acute myocardial infarction (AMI), arrhythmia, stroke and venous thromboembolism. The secondary outcomes were healthcare resource utilization in terms of discharge disposition, mean length of stay (LOS) (days) and hospital charges (US dollars).

Statistical analyses

Discharge weights were used to produce national estimates. Missing values were discarded from the final analyses. All temporal trends were assessed by linear-by-linear association test. The rates of diabetes decompensation (DKA/HHS) are represented as a percentage of total diabetes-related hospitalizations for each year whereas the mortality rates are calculated by dividing total in-hospital deaths by total cases of diabetes decompensation for each year. Correspondingly, age, gender, and race-stratified incidence and mortality rates were calculated by dividing total diabetes decompensation and in-hospital deaths by total diabetes and decompensated diabetes hospitalizations for a particular age, gender and race groups for each year, respectively. Baseline demographics, comorbidities, and outcomes were compared between diabetes hospitalizations with and without decompensation using the Chi-square test for categorical variables and Student’s t-test for continuous variables. We considered a two-tailed p-value ≤0.05 as statistical significance. We utilized Social Sciences software (SPSS), version 22.0 (IBM Corp., Armonk, NY, USA) for all analyses. 

## Results

Baseline characteristics and comorbidities

Of 56.7 million adult diabetes-related hospitalizations, 0.5 (0.9%) million of hospitalizations revealed in-hospital decompensation (DKA or HHS) from 2007 to 2014. The decompensation group often consisted younger (Mean±standard deviation (SD); 52±17 vs. 66±15 years; p<0.001), black (24.2% vs. 17.3%; p<0.001) and Hispanic (12.9% vs. 10.9%; p<0.001) patients as compared to those without decompensation. The decompensation cohort more often consisted of Medicaid (20.7% vs. 10.2%; p<0.001), self-pay (11.0% vs. 3.3%; p<0.001) or private insurance beneficiaries (23.6% vs. 20.0%; p<0.001) and belonged to lower household income quartile (36.9% vs. 33.0%; p<0.001). Acute decompensation was observed more often with non-elective admissions (95.3% vs. 80.2%; p<0.001) to large bed size and urban-teaching facilities (50.8% vs. 46.9%; p<0.001) in Southern (42.8% vs. 40.5%; p<0.001) and Western (19.4% vs. 16.8%; p<0.001) regions (Table 1).

**Table 1:** Baseline characteristics of diabetes hospitalizations with and without decompensation P-values ≤ 0.05 indicate statistical significance. DKA, Diabetic Ketoacidosis; HHS, Hyperosmolar Hyperglycemic State; HMO, Health Maintenance Organization; SD: Standard Deviation.

Variable	No Decompensation (N=56,258,977)		Decompensation (DKA or HHS) (N=516,805)		Overall Diabetes (N=56,775,782)		P
Age (years) at admission	N	%	N	%	N	%	
Mean age ± SD	66 ± 15		52 ± 17		66 ± 15		<0.001
18-44	4,474,607	(8.0%)	170,964	(33.1%)	4,645,570	(8.2%)	
45-64	19,775,407	(35.2%)	218,916	(42.4%)	19,994,323	(35.2%)	
≥65	32,008,964	(56.9%)	126,925	(24.6%)	32,135,889	(56.6%)	
Sex							0.436
Male	26,792,531	(47.6%)	246,397	(47.7%)	27,038,928	(47.6%)	
Female	29,461,137	(52.4%)	270,349	(52.3%)	29,731,486	(52.4%)	
Race							<0.001
White	32,641,661	(65.6%)	264,682	(57.0%)	32,906,343	(65.5%)	
African American	8,632,625	(17.3%)	112,221	(24.2%)	8,744,846	(17.4%)	
Hispanic	5,451,308	(10.9%)	59,875	(12.9%)	5,511,182	(11.0%)	
Asian or Pacific Islander	1,186,596	(2.4%)	9,455	(2.0%)	1,196,051	(2.4%)	
Native American	408,944	(0.8%)	4,654	(1.0%)	413,598	(0.8%)	
Other	1,466,771	(2.9%)	13,260	(2.9%)	1,480,032	(2.9%)	
Primary Expected Payer							<0.001
Medicare	35,709,505	(63.6%)	205,134	(39.8%)	35,914,639	(63.4%)	
Medicaid	5,720,247	(10.2%)	106,482	(20.7%)	5,826,730	(10.3%)	
Private including HMO	11,242,654	(20.0%)	121,428	(23.6%)	11,364,082	(20.1%)	
Self – Pay	1,850,048	(3.3%)	56,741	(11.0%)	1,906,789	(3.4%)	
No charge	213,112	(0.4%)	5,267	(1.0%)	218,379	(0.4%)	
Others	1,416,868	(2.5%)	20,237	(3.9%)	1,437,105	(2.5%)	
Median Household Income Quartile for Patients’ Zip code							<0.001
0-25^th^	18,147,238	(33.0%)	185,578	(36.9%)	18,332,815	(33.1%)	
26-50^th^	14,776,281	(26.9%)	132,919	(26.4%)	14,909,200	(26.9%)	
51-75^th^	12,523,515	(22.8%)	108,545	(21.6%)	12,632,060	(22.8%)	
76-100^th^	9,471,181	(17.2%)	75,700	(15.1%)	9,546,880	(17.2%)	
Type of Admission							<0.001
Non-elective	44,978,560	(80.2%)	491,436	(95.3%)	45,469,996	(80.3%)	
Elective	11,111,128	(19.8%)	24,034	(4.7%)	11,135,162	(19.7%)	
Bed size of Hospital							<0.001
Small	7,668,982	(13.7%)	63,292	(12.3%)	7,732,274	(13.7%)	
Medium	14,126,229	(25.2%)	133,669	(26.0%)	14,259,898	(25.3%)	
Large	34,151,909	(61.0%)	316,555	(61.6%)	34,468,464	(61.0%)	
Location/Teaching Status of Hospital							<0.001
Rural	7,093,756	(12.7%)	55,605	(10.8%)	7,149,362	(12.7%)	
Urban - non teaching	22,602,717	(40.4%)	197,111	(38.4%)	22,799,828	(40.4%)	
Urban - teaching	26,250,646	(46.9%)	260,800	(50.8%)	26,511,446	(47.0%)	
Hospital Region							<0.001
Northeast	10,662,641	(19.0%)	89,560	(17.3%)	10,752,201	(18.9%)	
Midwest	13,366,331	(23.8%)	105,499	(20.4%)	13,471,830	(23.7%)	
South	22,765,403	(40.5%)	221,439	(42.8%)	22,986,842	(40.5%)	
West	9,464,603	(16.8%)	100,307	(19.4%)	9,564,909	(16.8%)	

The decompensation group was found to have higher proportion of comorbidities such as deficiency anemia (25.8% vs. 23.3%), chronic blood loss anemia (1.7% vs. 2.7%), coagulopathy (8.0% vs. 4.9%), diabetes with chronic complications (26.9% vs. 18.8%), drug abuse (6.9% vs. 2.4%), alcohol abuse (7.5% vs. 2.9%), smoking (23.5% vs. 23.2%), marijuana abuse (1.9% vs. 0.5%), cocaine abuse (1.7% vs. 0.6%), and amphetamine abuse (0.6% vs. 0.1%), liver disorders (5.0% vs. 3.7%), fluid and electrolyte disorders (62.7% vs. 27.3%), other neurological disorders (8.8% vs. 8.1%) and psychosis (6.9% vs. 4.8%) as compared to those diabetic patients without decompensation (Table 2).

**Table 2:** Baseline comorbidities in diabetes-related hospitalizations with and without decompensation P-values ≤0.05 indicate statistical significance, DKA, Diabetic Ketoacidosis; HHS, Hyperosmolar Hyperglycemic State; MI, Myocardial Infarction; PCI, Percutaneous Coronary Intervention; CABG, Coronary Artery Bypass Grafting

Comorbidity	No Decompensation (N=56,258,977)		Decompensation (DKA or HHS) (N=516,805)		Overall Diabetes (N=56,775,782)		P	
	N	%	N	%	N	%	
Deficiency anemias	13,090,106	(23.3%)	133,256	(25.8%)	13,223,362	(23.3%)	<0.001
Rheumatoid arthritis/collagen vascular diseases	1,497,235	(2.7%)	7,800	(1.5%)	1,505,035	(2.7%)	<0.001
Chronic blood loss anemia	810,377	(1.4%)	8,768	(1.7%)	819,145	(1.4%)	<0.001
Congestive heart failure	8,322,450	(14.8%)	55,687	(10.8%)	8,378,137	(14.8%)	<0.001
Chronic pulmonary disease	13,078,014	(23.2%)	72,929	(14.1%)	13,150,943	(23.2%)	<0.001
Coagulopathy	2,757,738	(4.9%)	41,094	(8.0%)	2,798,832	(4.9%)	<0.001
Depression	6,690,600	(11.9%)	61,777	(12.0%)	6,752,377	(11.9%)	0.177
Diabetes, uncomplicated	45,681,704	(81.2%)	377,695	(73.1%)	46,059,399	(81.1%)	<0.001
Diabetes with chronic complications	10,577,273	(18.8%)	139,110	(26.9%)	10,716,383	(18.9%)	<0.001
Drug abuse	1,348,913	(2.4%)	35,594	(6.9%)	1,384,507	(2.4%)	<0.001
Alcohol abuse	1,641,121	(2.9%)	38,869	(7.5%)	1,679,990	(3.0%)	<0.001
Smoking	13,075,129	(23.2%)	121,624	(23.5%)	13,196,753	(23.2%)	<0.001
Marijuana abuse	287,515	(0.5%)	9,618	(1.9%)	297,134	(0.5%)	<0.001
Cocaine abuse	359,180	(0.6%)	8,933	(1.7%)	368,113	(0.6%)	<0.001
Amphetamine abuse	67,186	(0.1%)	3,029	(0.6%)	70,215	(0.1%)	<0.001
Hypertension	41,230,228	(73.3%)	281,275	(54.4%)	41,511,503	(73.1%)	<0.001
Hypothyroidism	7,794,548	(13.9%)	52,834	(10.2%)	7,847,382	(13.8%)	<0.001
Liver disease	2,073,814	(3.7%)	25,751	(5.0%)	2,099,564	(3.7%)	<0.001
Fluid and electrolyte disorders	15,330,626	(27.3%)	324,203	(62.7%)	15,654,829	(27.6%)	<0.001
Metastatic cancer	1,118,587	(2.0%)	6,430	(1.2%)	1,125,018	(2.0%)	<0.001
Other neurological disorders	4,561,379	(8.1%)	45,649	(8.8%)	4,607,029	(8.1%)	<0.001
Obesity	10,979,282	(19.5%)	68,225	(13.2%)	11,047,506	(19.5%)	<0.001
Paralysis	1,768,423	(3.1%)	14,960	(2.9%)	1,783,383	(3.1%)	<0.001
Peripheral vascular disorders	5,713,320	(10.2%)	38,172	(7.4%)	5,751,492	(10.1%)	<0.001
Psychoses	2,679,865	(4.8%)	35,757	(6.9%)	2,715,622	(4.8%)	<0.001
Pulmonary circulation disorders	1,531,356	(2.7%)	11,291	(2.2%)	1,542,647	(2.7%)	<0.001
Renal failure	12,812,496	(22.8%)	111,550	(21.6%)	12,924,045	(22.8%)	<0.001
Solid tumor without metastasis	1,218,692	(2.2%)	6,112	(1.2%)	1,224,805	(2.2%)	<0.001
Valvular disease	2,373,648	(4.2%)	12,959	(2.5%)	2,386,607	(4.2%)	<0.001
Weight loss	2,488,940	(4.4%)	47,405	(9.2%)	2,536,345	(4.5%)	<0.001
Dyslipidemia	25,420,113	(45.2%)	157,907	(30.6%)	25,578,021	(45.1%)	<0.001
Previous MI PCI CABG	11,084,254	(19.7%)	49,435	(9.6%)	11,133,689	(19.6%)	<0.001

In-hospital outcomes and trends in healthcare resource utilization and all-cause in-hospital mortality

Diabetes hospitalizations with decompensation demonstrated significantly higher in-hospital mortality (6.3% vs. 2.6%; p<0.001), average LOS (7.7 vs. 5.4 days; p<0.001), hospital charges ($65,904 vs. $42,889, p<0.001), and more frequent transfers to short-term hospitals (3.9% vs. 2.9%; p<0.001) in comparison with those without decompensation. The rates of AMI (10.4% vs. 4.8%; p<0.001), stroke (4.0% vs. 3.3%; p<0.001) and venous thromboembolism (3.5% vs. 2.6%; p<0.001) were substantially higher among diabetes hospitalizations with decompensation compared to those without (Table 3).

**Table 3:** Impact of decompensated diabetes on outcomes of diabetes-related hospitalizations P-values ≤0.05 indicate statistical significance. DKA: Diabetic Ketoacidosis; HHS: Hyperosmolar Hyperglycemic State; SNF: Skilled Nursing Facility; ICF: Intermediate Care Facility

Outcomes	No Decompensation (N=56,258,977)		Decompensation (N=516,805)		Overall Diabetes (N=56,775,782)		P
	N	%	N	%	N	%	
All-cause in-hospital mortality	1,470,386	(2.6%)	32,602	(6.3%)	1,502,988	(2.6%)	<0.001
Acute myocardial infarction	2,677,807	(4.8%)	53,555	(10.4%)	2,731,362	(4.8%)	<0.001
Arrhythmia	13,083,662	(23.3%)	85,867	(16.6%)	13,169,529	(23.2%)	<0.001
Stroke	1,864,884	(3.3%)	20,533	(4.0%)	1,885,417	(3.3%)	<0.001
Venous thromboembolism	1,443,948	(2.6%)	17,998	(3.5%)	1,461,946	(2.6%)	<0.001
Disposition							<0.001
Routine	31,497,907	(56.0%)	290,017	(56.1%)	31,787,924	(56.0%)	
Transfer to short-term hospital	1,609,749	(2.9%)	20,038	(3.9%)	1,629,787	(2.9%)	
Other transfers (SNF, ICF, other)	12,061,305	(21.5%)	94,495	(18.3%)	12,155,800	(21.4%)	
Home health care	9,017,517	(16.0%)	66,308	(12.8%)	9,083,825	(16.0%)	
Mean length of stay (days) Mean ± SD	5.4 ± 6.7		7.7 ± 9.5		5.5 ± 6.7		<0.001
Mean hospital charges	$ 42,889		$ 65,904		$ 43,098		<0.001

Among decompensated-related admissions, the all-cause mortality rates showed a non-significant declining trend from 6.7% in 2007 to 6.3% in 2014 (p_trend_= 0.134). The mean LOS remained static (8 days) during decompensation-related admissions from 2007 to 2014 whereas mean hospitalization charges steadily increased from $51,190 in 2007 to $75,865 in 2014 (p_trend_ <0.001).

Age-specific trends in decompensated diabetes and subsequent in-hospital mortality

**Figure 1:**
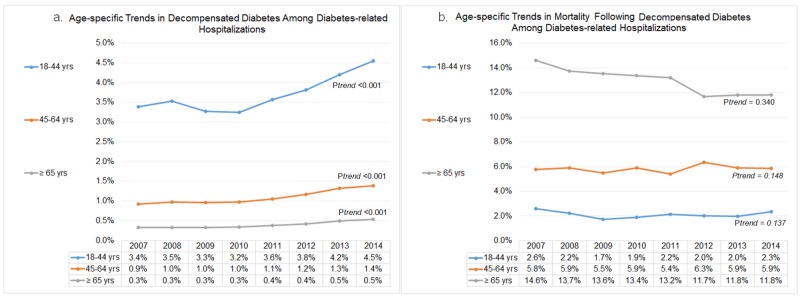
Age-specific trends in diabetes decompensation and subsequent in-hospital mortality

The overall rate of decompensated diabetes was highest among diabetics aged 18-44 (3.7%) years followed by 45-64 (1.1%) years and ≥65 (0.4%) years age group. However, advanced age group (≥65 years) showed a greater increments in the frequency of decompensated diabetics compared to younger diabetics, as noted by relative surges of 32.4% (from 3.4% to 4.5%), 55.6% (from 0.9% to 1.4%) and 66.7% (from 0.3% to 0.5%) in decompensated diabetes among 18-44 years, 45-64 years and ≥ 65 years age groups from 2007 to 2014, respectively (p_trend_<0.001) (Figure 1a). Older diabetics (≥65 years) had the highest in-hospital mortality (12.8%). However, we observed a non-linear trend in decompensation-related inpatient mortality among diabetics of all age groups from 2007 to 2014; 18-44 years (from 2.6% to 2.3%; p_trend_=0.137), 45-64 years (from 5.8% to 5.9%; p_trend_=0.148) and ≥65 years (from 14.6% to 11.8%; p_trend_=0.340) (Figure 1b).

Gender-specific trends in decompensated diabetes and subsequent in-hospital mortality

**Figure 2:**
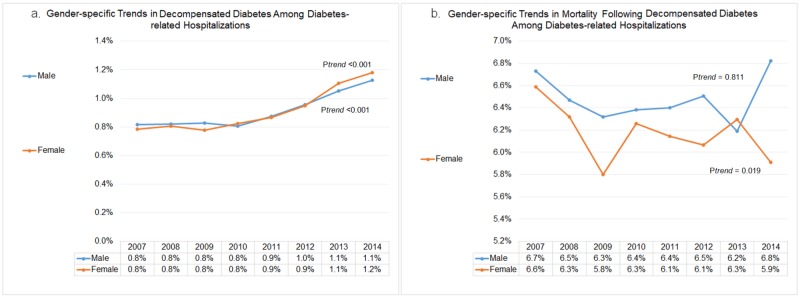
Gender-specific trends in decompensated diabetes and subsequent in-hospital mortality

The overall rate of decompensation among diabetes-related hospitalization was similar (0.9%) among male and female diabetics. Over the period of eight years, the rates of decompensation rose to 1.1% (p_trend_<0.001) in males and 1.2% (ptrend<0.001) in females, respectively; which corresponds to 37.5% and 50% relative increases in males and females, respectively (Figure 2a). All-cause in-hospital mortality among decompensated diabetic females declined significantly (from 6.6% in 2007 to 5.9% in 2014; p_trend_=0.019). However, there was no significant drop in in-hospital mortality among male diabetics with acute decompensation (from 6.7% in 2007 to 6.8% in 2014, p_trend_=0.811) (Figure 2b). 

Race-specific trends in decompensated diabetes and subsequent in-hospital mortality

**Figure 3:**
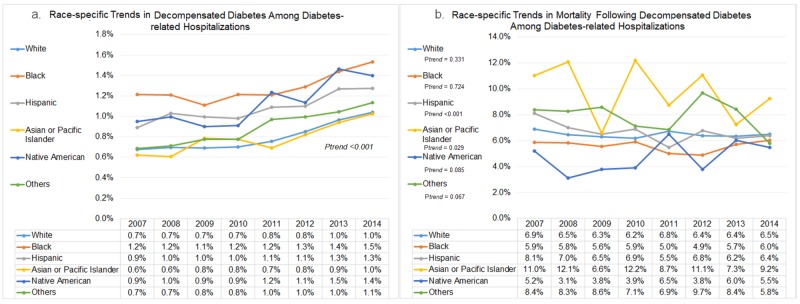
Race-specific trends in decompensated diabetes and subsequent in-hospital mortality

The rate of decompensated diabetes was highest among black patients with diabetes (1.3%). Native American (1.1%) and Hispanic (1.1%) had similar rates of decompensation, while white (0.8%) and Asian or Pacific Islander (0.8%) had the lowest rate of diabetes decompensation from 2007 to 2014. We observed significantly increasing trends in decompensated diabetes among all race groups between 2007 and 2014 (p_trend_<0.001) (Figure 3a). The in-hospital mortality among decompensation was highest among Asian or Pacific Islander (0.9%) from 2007 to 2014. There was a declining trend in the inpatient mortality among Asian or Pacific Islander (p_trend_=0.029) and Hispanic (p_trend_<0.001) patients with decompensated diabetes, whereas other race groups did not observe any significant trend in mortality over the study period (Figure 3b).

## Discussion

To our knowledge, this study is the largest study to date assessing the nationwide trends in decompensated diabetes across different age, gender and race groups, and its impact on the in-hospital outcomes among diabetics. The key findings of this population-based study are outlined as follows. The trends in decompensated diabetes have significantly increased over the years across all age groups (18-44, 45-64, ≥65 yrs), the greatest burden among younger diabetics. There was no significant decline in the mortality among decompensated diabetics across all age groups. Rising trends in DKA/HHS were determined in both sexes with a gender disparity in the trend of in-hospital mortality where declining trends were evident in female diabetics without a significant change in male diabetics. The higher rates of DKA/HHS hospitalizations among blacks and higher in-hospital mortality among Asians underline the notion of existing racial disparities in diabetes care. Patients with decompensated diabetes had a significantly high burden of all-cause in-hospital mortality, cardio-cerebrovascular events, and resource utilization.

This study reports a 50% relative increase (0.8% in 2007 to 1.2% in 2014) in decompensated diabetes (DKA/HHS) among diabetes-related hospitalization in the US from 2007 to 2014, which corresponds to a 54.9% increase reported by Benoit et al. from 2009-2014 and 59% increase reported by Desai et al. over the past decade [3, 11]. However, there was no significant decline in all-cause mortality owing to decompensated diabetes from 2007 to 2014. The overall increasing trend in decompensation could just be a reflection of a dramatic four-fold increase (from 5.5 million in 1980 to 22 million in 2014) in the diabetes prevalence in the US [12]. Furthermore, it could also be a reflection of shifting demographics and associated precipitating factors for diabetes and decompensation. To effectively understand these shifting trends, we performed a subgroup analysis of trends stratified by age, gender and race groups.

Our findings suggest that younger age group (18-44 years) witnessed the highest rates of DKA/HHS related hospitalizations between 2007 and 2014 consistent with data reported from previous epidemiological studies [3, 13]. Benoit et al. reported the highest DKA hospitalization among younger patients (44.3 per 1,000), while it was low among elderly diabetics (less than 2 per 1,000) [11]. Several factors can influence the age-specific trends in decompensated diabetes-related hospitalizations and outcomes. Firstly, the impaired effective level and action of insulin [5]. The dramatic increase in the prevalence of obesity, over the decade, in the US has drastically increased the burden of insulin resistance and make this population prone towards DKA/HHS [14]. Furthermore, the rising prevalence of ketosis-prone diabetes among type 2 obese diabetics also bears a high risk for DKA recurrence after insulin discontinuation after the resolution of ketoacidosis [15]. Furthermore, the rising burden of the use of recreational drugs, evident in our decompensated diabetic cohort, could be a potential factor for the rising trends in DKA/HHS and subsequent poor cardiovascular outcomes. Studies suggest a high burden (15-20%) of substance abuse among adults with DKA-related hospitalizations [16-17]. Rising trends in drug abuse especially cocaine and marijuana have been concerning as these substances have the potential to increase the odds of MI, arrhythmia, and stroke as noted in prior studies [18-21]. Such practices make these patients prone towards discontinuation of insulin therapy, dietary transgressions, behavioral issues, stress and could alter metabolism leading to an increased risk of decompensation [8, 16].

We observed an increase from 0.3% to 0.5% in the rate of decompensated diabetes-related hospitalizations among older diabetics (≥65 years). Elderly patients are prone towards prolonging immobilization and has altered thirst response hampering their hydration status and increases the risk of HHS [4, 22]. This is evident from a significantly high burden of fluid and electrolyte disturbances among diabetics with decompensation in our study. Moreover, hyperglycemia can derange hydration and platelet aggregation hampers endothelium-derived nitric oxide levels leading to a hypercoagulable state [23-24]. Hypercoagulable states increase the risk of coagulopathy, cardio-cerebrovascular events, and venous thromboembolism as evident by a high proportion of these events in our study [23].

The low rate of decompensated diabetes among elderly diabetics was associated with high rates of in-hospital mortality as compared to young age, which reflects the increased severity of decompensation and comorbid disease burden in older diabetics. HHS most commonly occurs among elderly patients and accounts for less than 1% of diabetes admissions [4] whereas DKA is more common in the younger age group [5]. However, HHS has been reported to pose 10 times higher risk of mortality than DKA [8, 25]. We did not observe any significant decline in the mortality across any age group of decompensated diabetics, which is a concerning result and need further exploration.

The gender-specific rate of decompensated diabetes was similar among male and female patients. However, by 2014 there was a 50% increase in hospitalization rate among females whereas for males the relative increase was around 37.5%. Desai et al. also reported a similar increase in DKA-related discharges among females as compared to males over a period of 10 years in the US hospitals [3]. All-cause in-hospital mortality in diabetics with decompensation showed a significantly declining trend only among females with a varying non-linear trend among males. Women reluctance towards self-care and prioritization of family health over own and intentional omission of insulin have been reported to be the major reasons behind such disparity [26]. However, despite high-risk behavior among female decline in the mortality need further evaluation for the factors governing such changes.

In the present study, an increase in the decompensated diabetes-related hospitalization was observed across all the race groups. Black patients had the highest burden of decompensated diabetes with a 25% relative increase (from 1.2% to 1.5%) from 2007 to 2014 without any significant change in mortality over the study period. High burden of obesity, use of illicit drugs, poor insulin compliance could be a potential reason for the high rate of decompensated diabetes among blacks [17]. Interestingly, Asians had the lowest burden of decompensation in 2007 and 2014 with the highest relative surge of 66.66% (from 0.6% to 1%) from 2007 to 2014. Despite the lowest burden of decompensated diabetes, Asians suffered the highest mortality rate both in 2007 and 2014 with an encouraging decline of 16.36% (from 11% to 9.2%) from 2007 to 2014. Furthermore, we noticed the highest proportion of diabetes and decompensated diabetes-related hospitalizations in the Southern region hospitals. CDC report also suggests a Southern regional predominance of DKA especially among obese African Americans [12]. 

In addition to the increased inpatient mortality, the decompensated diabetics were found to have a higher proportion of in-hospital MI, stroke and venous thromboembolism events, which may further lead to worse outcomes [10]. Interestingly, the stroke rate was higher in the decompensated group even with a lower frequency of arrhythmia compared to those without decompensation. That suggests that diabetes decompensation may independently increase stroke events in diabetes patients. A higher mean hospital stay and hospitalization charges were reflective of DKA/HHS-related healthcare burden.

Limitations

This study bears a few limitations. The NIS is an administrative database and utilized the ICD-9 CM coding system for discharges, hence it may under or overestimate the diagnoses at times. The NIS follows visit-based approach rather than a patient-based approach for data collection, therefore, it is possible that one patient may have been captured multiple times. The analysis was not stratified by type of diabetes. In addition, the temporal trends in decompensated diabetes are governed by multiple factors including varying demographic landscapes of diabetics, access to health care, medication compliance, socioeconomic status, and varying degree of insulin resistance, which should be considered while inferring results. There is a lack of any longitudinal follow-up data. This study determines the temporal trends in decompensated diabetes but causative role of any demographics on prevalence or outcomes cannot be established. The exact cause of in-hospital mortality cannot be ascertained from the NIS dataset. However, the large sample size and weighted discharges empowered us to obtain comprehensive national estimates in the US similar to our prior studies regarding healthcare resource utilization and outcomes trends [27-30]. These limitations have insignificantly impacted the important outcomes drawn from this retrospective study.

## Conclusions

In this nationwide population-based analysis in the US, the burden of decompensated diabetes (DKA/HHS) among hospitalized diabetics has shown an increasing trend from 2007 to 2014. Despite improved screening measures and healthcare delivery in recent times, the rising trends in the decompensated diabetes rate, and its gender and racial variability are of great concern. These concerning trends warrant reinforced preventive steps to control precipitant factors towards diabetes decompensation and safeguard the US health care infrastructure from resultant poor outcomes and consequent higher healthcare cost.
